# Preoperative Folate Receptor-Positive Circulating Tumor Cells Are Associated With Occult Peritoneal Metastasis and Early Recurrence in Gastric Cancer Patients: A Prospective Cohort Study

**DOI:** 10.3389/fonc.2022.769203

**Published:** 2022-03-29

**Authors:** Ci Dian Dan Zeng, Cheng Cheng Jin, Chun Gao, Ai Tang Xiao, Yi Xin Tong, Sheng Zhang

**Affiliations:** Department of Gastrointestinal Surgery, Tongji Hospital, Tongji Medical College, Huazhong University of Science and Technology, Wuhan, China

**Keywords:** gastric cancer, circulating tumor cell, folate receptor, recurrence-free survival, peritoneal metastasis

## Abstract

**Background:**

The aim of this study is to explore the clinical feasibility of detecting folate receptor-positive circulating tumor cells (FR+ CTCs) for predicting peritoneal metastasis and short-term outcome in gastric cancer patients.

**Methods:**

This is a prospective, single-center, observational study. We applied ligand-targeted enzyme-linked polymerization method to detect preoperative FR+ CTC levels in peripheral blood. We evaluated the diagnostic value of FR+ CTCs and other biomarkers in predicting peritoneal metastasis. Prognostic factors for recurrence-free survival (RFS) were investigated in univariate and multivariate analyses.

**Results:**

A total of 132 patients with gastric cancer and 9 patients with benign disease were recruited. Gastric cancer patients had a significantly higher CTC level compared to that of patients with benign disease (p < 0.01). Combined model including CTC level and other biomarkers presented high sensitivity (100%) and moderate specificity (59.3%) in predicting peritoneal metastasis. Univariate analysis revealed that decreased serum prealbumin, decreased peripheral lymphocyte count, FR+ CTCs, carcinoembryonic antigen (CEA), carbohydrate antigen 19-9 (CA19-9), and lymph node metastasis were significantly associated with shorter RFS. FR+ CTC level [≥12.6 folate units (FU)/3 ml, hazard ratio (HR) = 6.957, p = 0.005] and CA19-9 (>34 ng/ml, HR = 3.855, p = 0.037) were independent prognostic factors in multivariate analysis.

**Conclusions:**

Our findings for the first time suggested the diagnostic value of preoperative CTC levels in predicting peritoneal metastasis in gastric cancer. Moreover, the FR+ CTC level could be a novel and promising prognostic factor for the recurrence of gastric cancer in patients who underwent surgery.

**Clinical Trial Registration:**

Chinese Clinic Trial Registry, identifier ChiCTR2100050514.

## Introduction

Globally, gastric cancer (GC) is a common malignancy and a leading cause of cancer death. Curative operation together with perioperative chemotherapy was the standard treatment for advanced gastric cancer patients (1–4). Although comprehensive treatment strategies have been developed, the recurrence rate of gastric cancer is relatively high, and the long-term prognosis is poor. Specially, a proportion of patients with gastric cancer experienced recurrence within 1 year after surgery. The prognosis of gastric cancer patients who suffered from early recurrence is very poor, which urges us to identify risk factors to predict early recurrence in patients with gastric cancer after radical gastrectomy (5, 6).

Circulating tumor cells (CTCs) are the cells shed from primary or metastatic tumors and subsequently entered the circulation. CTCs were reported to be closely related to tumor development, metastasis, or recurrence (7–9). Studies have shown that CTCs could be a significant prognostic factor in various malignancies such as metastatic colorectal cancer, pancreatic cancer, prostate cancer, and non-small cell lung cancer (NSCLC) (10–13). In addition, detection of CTCs in peripheral blood can also assist in the diagnosis of solitary pulmonary nodules and predict treatment response in NSCLC (14–16).

Folate receptor (FR) is highly expressed on the surface of the cell membrane in various types of malignant tumors mediating cellular folate transportation (17). FRα was the most common isoform that was aberrantly overexpressed in cancer tissue compared to normal tissue. In the circulation, detection of folate receptor-positive (FR+) cells could serve as a simple and non-invasive method to identify CTCs in peripheral blood (18, 19). Ligand-targeted enzyme-linked polymerization was applied to detect FR+ CTCs in peripheral blood and has shown diagnostic value in early detection of various malignancies (20, 21). Previous studies proved that detection of FR+ CTCs has both diagnostic and prognostic value in lung cancer and pancreatic cancer (11–16, 22). However, the clinical diagnostic and predictive value of CTCs in gastric cancer patients has not been studied. In this study, we aimed to investigate the diagnostic value of CTCs for predicting peritoneal metastasis (PM) in gastric cancer patients. Furthermore, we attempt to explore the prognostic value of preoperative CTC levels for recurrence-free survival (FRS) in gastric cancer patients.

## Methods

### Study Design and Participants

We conducted a prospective observational study in a single institution. From April 2020 to April 2021, a total of 132 gastric cancer patients who were scheduled to receive laparoscopic gastrectomy according to the Japanese Gastric Cancer Treatment Guidelines (23) were included in our study. The inclusion criteria were as follows: 1) patients aged from 18 to 80 years; 2) patients underwent operation as laparoscopic gastrectomy lymph node dissection with the postoperative histopathological diagnosis confirmed as gastric adenocarcinoma; 3) patients without a previous history of other malignancies; 4) patients without neoadjuvant chemotherapy. This study was approved by the institutional medical ethics committee (TJH20200401) and registered at the Chinese Clinic Trial Registry (ChiCTR2100050514), with all aspects in this study complying with the 1964 Helsinki Declaration and later versions. All participants provided a written informed consent. We present this article in accordance with the STrengthening the Reporting of OBservational studies in Epidemiology (STROBE) checklist **(** **Supplementary Table 1** **) (** 24 **).**

### Data Collection and Folate Receptor-Positive Circulating Tumor Cell Analysis

Peripheral blood samples were collected immediately after admission. CTCs were enriched and quantified using the CytoploRare Kit (Genosaber Biotech, Shanghai, China) according to the manufacturer’s instructions. In brief, 3 ml peripheral blood was first collected to enrich CTCs by lysis of erythrocytes followed by immunomagnetic depletion of white blood cells. FR+ CTCs were then quantified by ligand-targeted enzyme-linked polymerase chain reaction (LT-PCR). The level of FR+ CTCs in each sample was calculated based on the calibration curve generated with the standard reference provided in the kit. The unit of CTC level was denoted as “FU” indicating folate units (FU)/3 ml of blood. The primer sequences used in PCR were as follows: detection probe, 5’–CTCAA CTGGT GTCGT GGAGT CGGCA ATTCA GTTGA GGGTT CTAA–3’; forward primer, 5’–TATGA TTATG AGGCA TGA–3’; reverse primer, 5’–GGTGT CGTGG AGTCG–3’; TaqMan probe, 5’–FAM–CAGTT GAGGG TTC–MGB–3’ (15, 19).

We prospectively collected the following demographic and clinical data for analysis: 1) Demographic characteristics such as age, gender, body mass index (BMI), smoking, and alcohol; 2) Laboratory characteristics such as albumin, prealbumin, serum tumor markers such as carcinoembryonic antigen (CEA), carbohydrate antigen 19-9 (CA19-9), and CA72-4; 3) Clinical characteristics such as invasion depth (T), presence of lymph node metastases (N), and tumor-node-metastasis stage (TNM); 4) Perioperative characteristics such as operation type, length of hospital stay, and postoperative complication [according to Clavien–Dindo criteria (25)].

### Follow-Up and Study Outcome

Patients were regularly followed up every month in the first 12 months after surgery and then every 3–6 months thereafter. Follow-up investigations included physical examination, blood tests (such as complete blood count, liver functions, CEA, CA19-9, etc.), chest radiography, abdominal contrast-enhanced computerized tomography scanning, and annual endoscopic examination.

The primary endpoint was occult peritoneal metastasis (OPM). OPM was diagnosed during intended radical operation with no radiological evidence of PM before surgery. Pathological examination of PM was required for confirmed diagnosis. The secondary endpoint of this study was tumor recurrence. Tumor recurrence was defined as the diagnosis of tumor based on radiologic finding with or without biopsies. The RFS was defined as the time interval between the date of gastrectomy to the date of recurrence or last follow-up without recurrence or metastasis.

### Statistical Analyses

We presented continuous variables as mean [standard deviation (SD)]/medians (range) and analyzed with Student’s t-test or Mann–Whitney U test. We presented categorical variables as whole numbers and percentages and compared using chi-square test or Fisher’s exact test. The Youden index was calculated using the receiver operating characteristic (ROC) curve to determine the most efficient cutoff point of each risk factor for the diagnosis of PM. The combined model was constructed by logistic regression formula including the individual risk factors. We applied R software pROC package to display and analyze the ROC curve. We used the ROC curve to evaluate sensitivity (true positive rate/(true positive rate + false negative rate) ×100%) and specificity (true negative rate/(true negative rate + false positive rate) ×100%) of PM prediction of each risk factor and the combined model. ROCs were compared using the DeLong test (26). Univariate and multivariate logistic regression analyses were used to analyze the potential risk factors for PM.

The cutoff value of FR+ CTC level to stratify the study population into different prognostic groups were determined *via* maximally selected rank statistics (R package “maxstat” https://cran.r-project.org). RFS were analyzed using the Kaplan–Meier method, and differences were assessed by the log-rank test. The univariate Cox proportional regression was used to evaluate potential risk factors affecting RFS. Only factors with p-value <0.1 in univariate analysis were included in the final multivariate analysis model. Multivariate Cox regression was employed to identify independent risk factors on RFS and OS with backward stepwise method. All p-values were reported as two-sided with a significance level of 0.05. All statistical tests were performed in SPSS version 24.0 (IBM, NY, USA), and graphing was performed by GraphPad Prism version 9.0 software.

## Results

### Demographic and Clinical Characteristics of Included Patients

**Figure 1:**
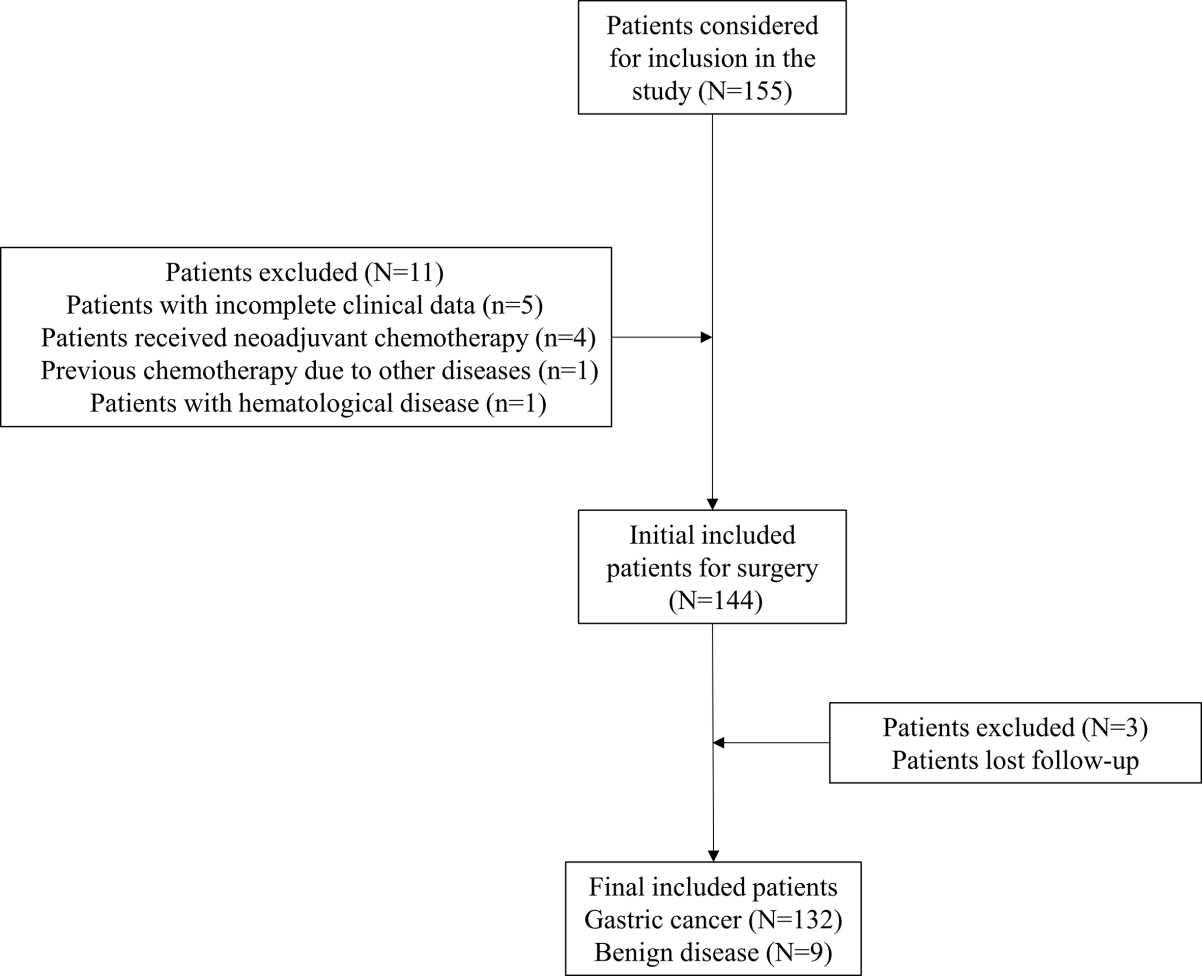
Flowchart of included patients.

After screening based on inclusion and exclusion criteria, 132 gastric cancer patients who underwent laparoscopic gastrectomy were included in the final study. Nine patients with benign diseases who underwent laparoscopic gastrectomy were also included as control. The flowchart of patients included was demonstrated as **Figure 1**. Demographic and clinical parameters were summarized in **Table 1**. In general, the mean age of included patients was 59.2 ± 10.5 years old. Ninety-one (68.9%) patients were men, and 41 (31.1%) were women. Clinical and pathological examinations indicated that 27 (20.5%), 32 (24.2%), 54 (40.9%), and 19 (14.4%) patients were with stage I, II, III, and IV disease, respectively. In addition, 105 (82.0%) patients underwent curative operation, while 23 (18.0%) patients received palliative or no (or open/close) operation. The median duration of postoperative hospital stay was 9 days [interquartile range (IQR): 8–10]. The median follow-up time was 6.2 months (IQR: 4.6–7.5).

**Table 1:** The demographic and laboratory characteristics of all patients (n = 132).

Variables	All Patients
**Demographic**	
Age, mean (SD), years	59.2 ± 10.5
Gender	
Men	91 (68.9%)
Women	41 (31.1%)
Smoking history	
No	100 (75.8%)
Yes	32 (24.2%)
Alcohol	
No	109 (82.6%)
Yes	23 (17.4%)
Hypertension	
No	98 (74.2%)
Yes	34 (25.8%)
Diabetes	
No	117 (88.6%)
Yes	15 (11.4%)
**Laboratory**	
Albumin (g/L), mean (SD)	38.9 ± 4.0
Prealbumin (mg/L), mean (SD)	209.8 ± 41.8
CEA	
Normal (≤5 ng/ml)	95 (72.0%)
Elevated (>5 ng/ml)	37 (28.0%)
CA19-9	
Normal (≤34 ng/ml)	102 (82.3%)
Elevated (>34 ng/ml)	22 (17.7%)
CA72-4	
Normal (≤6.9 ng/ml)	97 (78.2%)
Elevated (>6.9 ng/ml)	27 (21.8%)
**Clinicopathological**	
T stage	
1	16 (14.0%)
2	18 (15.8%)
3	45 (39.5%)
4	35 (30.7%)
Lymph node metastasis	
No	59 (52.7%)
Yes	53 (47.3%)
Distant metastasis	
No	113 (85.6%)
Yes	19 (14.4%)
TNM stage	
I	27 (20.5%)
II	32 (24.2%)
III	54 (40.9%)
IV	19 (14.4%)
Operation	
No or open/close	9 (7.0%)
Palliative	14 (10.9%)
Curative	105 (82.0%)
Postoperative duration of hospital stay, days,	9 (8, 10)
Postoperative complication	
No	119 (90.1%)
Grade 1&2	12 (9.1%)
Grade 3	1 (0.8%)

CEA, carcinoembryonic antigen; CA19-9, carbohydrate antigen 19-9; CA72-4, carbohydrate antigen 72-4; IQR, interquartile range; TNM, tumor-node-metastasis.

### Correlation Between Folate Receptor-Positive Circulating Tumor Cell Levels and Clinicopathological Features

**Figure 2:**
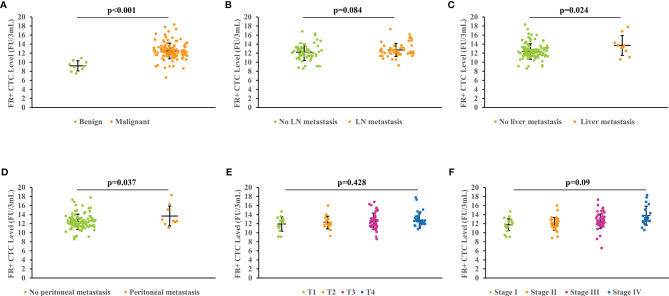
The comparison of FR+ CTCs level based on clinical characteristics **(A)** Benign and malignant diseases; **(B)** Lymph node (LN) metastasis; **(C)** liver metastasis; **(D)** peritoneal metastasis; **(E)** T stage; **(F)** TNM stage. FR+CTCs, folate receptor-positive circulating tumor cells; T1, tumor invades the lamina propria, muscularis mucosae or submucosa; T2, tumor invades the muscularis propria; T3, tumor penetrates the subserosal connective tissues without invasion of the visceral peritoneum; T4, tumor invades the serosa or adjacent structures; TNM, tumor-node-metastasis.

The Kolmogorov–Smirnov normality test showed that FR+ CTC levels in the gastric cancer group (p = 0.089) and benign disease group (p = 1.000) were normally distributed. Therefore, we present FR+ CTCs levels as mean ± SD. Compared to benign disease (9.19 ± 1.13), the FR+ CTC level was significantly higher in gastric cancer patients (12.49 ± 1.78) (p < 0.001) (**Figure 2A**).

We explored the correlation between FR+ CTC levels and clinicopathological features. We found no significant correlation between FR+ CTC level and clinical parameters including age, gender, history of smoking, and alcohol. Interestingly, we discovered that the FR+ CTC level was significantly correlated with advanced clinical stage (**Figure 2**). Patients with PM or liver metastasis had a higher FR+ CTC level than that of their counterparts, with 13.68 ± 2.36 vs. 12.39 ± 1.71 (p = 0.037) and 13.70 ± 2.24 vs. 12.38 ± 1.71 (p = 0.024), respectively (**Figure 2**). We also found a positive correlation between FR+ CTC levels and serum tumor biomarkers CEA (p = 0.024) and CA19-9 (p = 0.006). Other details were summarized in **Table 2**. In addition, the details of the pathologies were listed in **Supplementary Table 2**.

**Table 2:** CTC level in patients grouped according to different clinical characteristics (n = 132).

Variables	N	CTC level, mean±SD, FU/3mL	p-value
Age, y			0.308
<60	63	12.31 ± 1.47	
≥60	69	12.64 ± 2.02	
Gender			0.696
Male	91	12.52 ± 1.87	
Female	41	12.40 ± 1.56	
Disease			**<0.001**
Benign disease	9	9.19 ± 1.13	
Gastric cancer	132	12.49 ± 1.78	
Smoking			0.525
No	100	12.54 ± 1.87	
Yes	32	12.30 ± 1.50	
Alcohol			0.214
No	109	12.58 ± 1.88	
Yes	23	12.06 ± 1.21	
CEA			**0.024**
Normal (≤5 ng/mL)	95	12.24 ± 1.81	
Elevated (>5 ng/mL)	37	13.04 ± 1.62	
CA19-9			**0.006**
Normal (≤34 ng/mL)	102	12.24 ± 1.78	
Elevated (>34 ng/mL)	22	13.44 ± 1.61	
CA72-4			
Normal (≤6.9 ng/mL)	97	12.31 ± 1.88	
Elevated (>6.9 ng/mL)	27	13.05 ± 1.36	0.075
Lymph node metastasis			0.084
No	59	12.14 ± 1.87	
Yes	53	12.72 ± 1.49	
Peritoneal metastasis			**0.037**
No	123	12.39 ± 1.71	
Yes	9	13.68 ± 2.36	
Liver metastasis			**0.024**
No	122	12.38 ± 1.71	
Yes	10	13.70 ± 2.24	
TNM stage			**0.001**
I	19	11.89 ± 1.43	
II	32	11.95 ± 1.43	
III	54	12.54 ± 1.73	
IV	19	13.77 ± 2.16	

For p-value: Boldface type indicates significant difference. CEA, carcinoembryonic antigen; CA19-9, Carbohydrate antigen 19-9; CA72-4, Carbohydrate antigen 72-4; SD, standard deviation; TNM, tumor-node-metastasis.

### Diagnostic Value of Folate Receptor-Positive Circulating Tumor Cells for Peritoneal Metastasis in Gastric Cancer

**Figure 3:**
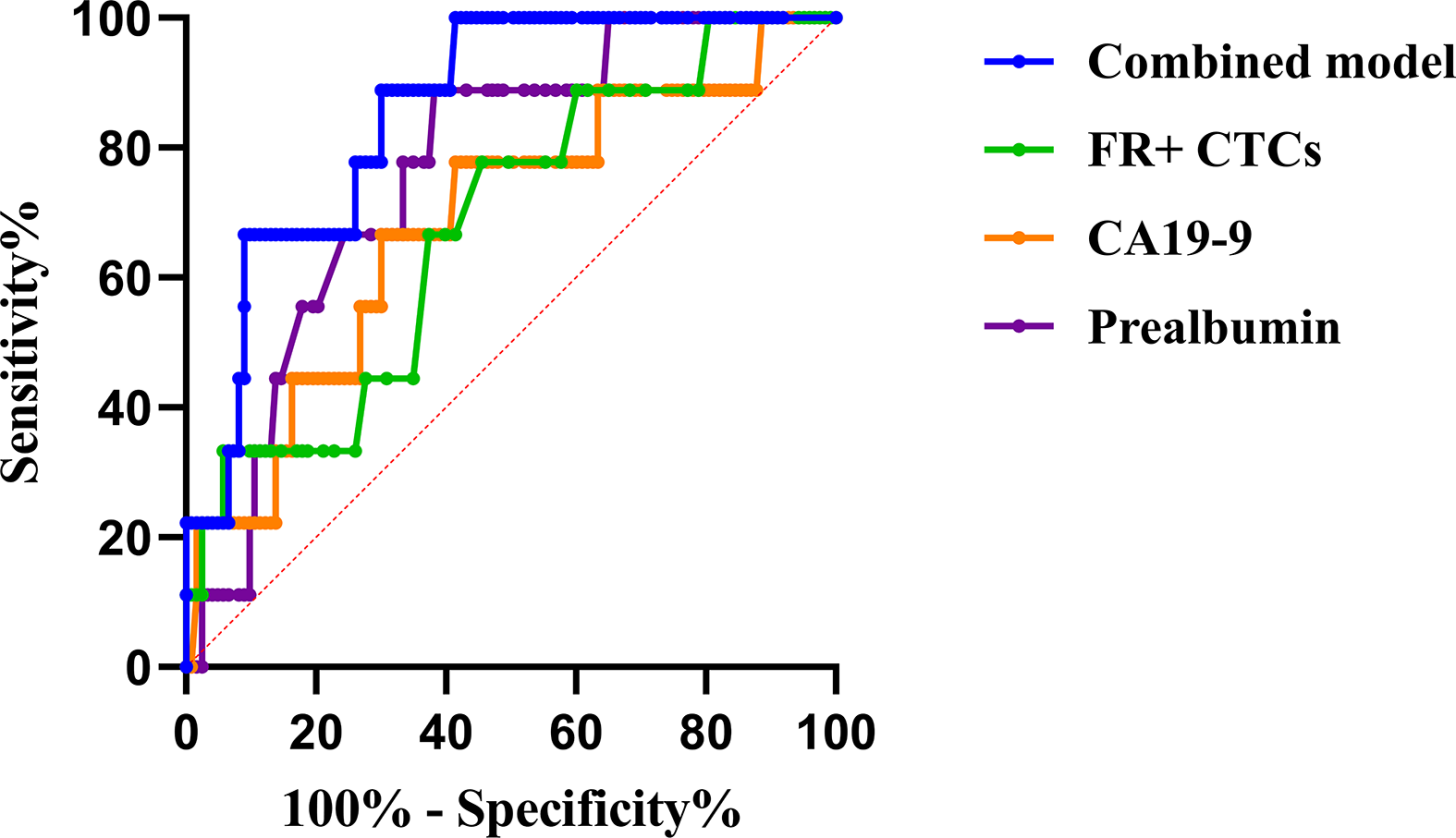
The ROC curve of potential risk factors and combined model for peritoneal metastasis. * ROC, receiver operating characteristic; FR+CTCs, folate receptor-positive circulating tumor cells; CA19-9, carbohydrate antigen 19-9.

We applied ROC curves to compare the diagnostic efficiencies of FR+ CTC levels and other biomarkers for PM. The optimal FR+ CTC level cutoff value for predicting patients with PM was 12.3 FU/3 ml, with a sensitivity of 77.8% and a specificity of 54.5%, and the AUROC was 0.68 (95% CI, 0.51–0.85, p = 0.07). We also found that CA19-9 and prealbumin showed diagnostic values for PM, with AUROC of 0.67 (95% CI, 0.49–0.86, p = 0.08) and 0.77 (95% CI, 0.64–0.70, p = 0.01), respectively (**Table 3**). The combination model (include FR+ CTCs, CA19-9, prealbumin, and peripheral lymphocyte count) showed superior efficiencies in predicting PM, with a sensitivity of 100% and a specificity of 59.3%, and the AUROC was 0.86 (95% CI, 0.76–0.95, p < 0.01) (**Figure 3**). Univariate logistic regression analyses revealed that FR+ CTC level (p = 0.025), CA19-9 (p = 0.097), CA72-4 (p = 0.045), and prealbumin (p = 0.023) were significantly associated with PM. However, the multivariate analysis showed that only the FR+ CTC was an independent risk factor for PM (**Supplementary Table 3**).

**Table 3:** Diagnostic values of FR+ CTCs and other biomarkers risk factors for peritoneal metastasis.

Variable	Sensitivity%	Specificity%	AUC (95%CI)	P value
FR+ CTCs	77.8%	54.5%	0.68 (0.51-0.85)	**0.07**
CEA	33.3%	87.8%	0.55 (0.33-0.77)	0.67
CA19-9	44.4%	82.6%	0.67 (0.49-0.86)	**0.08**
CA72-4	44.4%	86.1%	0.59 (0.38-0.82)	0.35
Albumin	66.7%	43.1%	0.62 (0.46-0.77)	0.25
Prealbumin	88.9%	60.2%	0.77 (0.64-0.70)	**0.01**
Peripheral lymphocyte count	66.7%	66.7%	0.66 (0.48-0.85)	**0.11**
Combined predictive model	100%	59.3%	0.86 (0.76-0.95)	**<0.01**

For p-value: Boldface type indicates biomarker included in the final predictive model. AUC, area under curve; FR+ CTCs, Folate receptor positive circulating tumor cells; CEA, carcinoembryonic antigen; CA19-9, Carbohydrate antigen 19-9; CA72-4, Carbohydrate antigen 72-4.

### Prognostic Value of Folate Receptor-Positive Circulating Tumor Cells for Recurrence-Free Survival in Gastric Cancer

**Figure 4:**
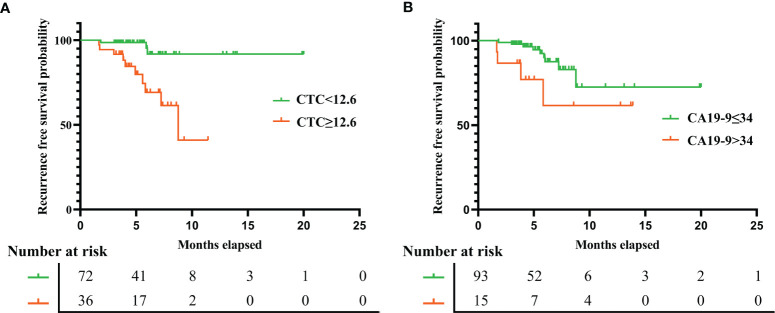
The recurrence free survival rate in gastric cancer patients based on preoperative FR+ CTCs level **(A)** and serum CA19-9 level **(B)**. FR+CTCs, folate receptor-positive circulating tumor cells; CA19-9, carbohydrate antigen 19-9.

The median duration of RFS was 5.2 months (IQR, 3.8–7.1). The median duration of RFS in the FR+ CTC group and FR- CTC group was 4.8 months (IQR, 3.8–7.2) and 5.4 months (IQR, 3.8–7.1) respectively. The details of follow-up were listed in **Supplementary Table 4**. Univariate Cox regression analysis revealed that decreased serum prealbumin [<210 mg/L, hazard ratio (HR) = 4.107, p = 0.067], decreased peripheral lymphocyte count (<1.65 * 10^9^/L, HR = 8.045, p = 0.046), FR+ CTC level (≥12.6 FU/3 ml, HR = 6.898, p = 0.003), CEA level (>5 ng/ml, HR = 4.82, p = 0.006), CA19-9 level (>34 ng/ml, HR = 3.084, p = 0.07), and lymph node metastasis (HR = 5.002, p = 0.015) were significantly associated with shorter RFS (**Figure 4**). We further categorized patients into three groups: Group A: patients without elevated FR+ CTCs (≥12.6 FU/3 ml) and elevated CA19-9 (>34 ng/ml); Group B: patients with either elevated FR+ CTCs (≥12.6 FU/3 ml) or elevated CA19-9 (>34 ng/ml); Group C: patients with both elevated FR+ CTCs (≥12.6 FU/3 ml) and elevated CA19-9 (>34 ng/ml). As demonstrated in **Supplementary Figure 1**, patients in group C had a significant shorter RFS compared to those in group A and group B (p < 0.01).

Multivariate analysis using Cox proportional hazards model demonstrated that the independent prognostic factors of RFS were FR+ CTC level (≥12.6 FU/3 ml, HR = 6.957, p = 0.005) and CA19-9 level (>34 ng/ml, HR = 3.855, p = 0.037), suggesting that the preoperative CTC level could serve as a novel and valuable biomarker to predict short-term recurrence in gastric cancer patients after surgery. The details of univariate and multivariate analysis were listed in **Table 4**.

**Table 4:** Univariate and multivariate Cox regression analyses predicting recurrence in gastric cancer patients after operation.

Variable	Univariate HR (95%CI)	p	Multivariate HR (95%CI)	p
Age				
<60y	1 (Ref)	0.491		
≥60y	1.479 (0.482-4.536)			
Gender				
Male	1 (Ref)	0.224		
Female	0.446 (0.121-1.639)			
Smoking				
No	1 (Ref)	0.128		
Yes	3.624 (0.716-13.018)			
Alcohol				
No	1 (Ref)	0.131		
Yes	3.284 (0.737-12.823)			
Albumin				
≥35g/L	1 (Ref)	0.342		
<35g/L	1.699 (0.570-5.063)			
Prealbumin				
≥210mg/L	1 (Ref)	**0.067**	N/A	NS
<210mg/L	4.107 (0.908-18.577)			
Peripheral lymphocyte count				
≥1.65*10^9^/L	1 (Ref)	**0.046**	N/A	N/A
<1.65*10^9^/L	8.054 (1.040-62.379)			NS
FR+ CTCs level				
<12.6 FU/3mL	1 (Ref)	**0.003**	1 (Ref)	**0.005**
≥12.6 FU/3mL	6.898 (1.896-25.096)		6.957 (1.811-26.727)	
CEA				
≤5ng/mL	1 (Ref)	**0.006**	N/A	NS
>5ng/mL	4.82 (1.563-14.867)			
CA19-9				
≤34ng/mL	1 (Ref)	**0.07**	1 (Ref)	**0.037**
>34ng/mL	3.084 (0.912-10.431)		3.855 (1.082-13.732)	
T stage				
T1/T2	1 (Ref)	0.287		
T3/T4	2.276 (0.501-10.333)			
Lymph node metastasis				
No	1 (Ref)	**0.015**	N/A	NS
Yes	5.002 (1.363-18.358)			
Signet-ring cell carcinoma				
No	1 (Ref)	0.146		
Yes	2.412 (0.735-7.919)			

For p-value: Boldface type indicates significant difference. HR, hazard ratio; FR+ CTCs, Folate receptor positive circulating tumor cells; CEA, carcinoembryonic antigen; CA19-9, Carbohydrate antigen 19-9; N/A, not applicable; NS, no significant difference. T1, tumor invades the lamina propria, muscularis mucosae or submucosa; T2, tumor invades the muscularis propria; T3, tumor penetrates the subserosal connective tissues without invasion of the visceral peritoneum; T4, tumor invades the serosa or adjacent structures.

## Discussion

The mainly revealed result in this investigation was that preoperative FR+ CTC level was associated with OPM and shorter RFS in gastric cancer patients. To date, CTCs have been applied as a non-invasive technique in early diagnosis, outcome prediction, and treatment evaluation in various malignancies. In this study, we reported a prospective observational study to investigate the clinical value of FR+ CTCs in predicting PM and short-term RFS in gastric cancer patients. Our results for the first time showed that FR+ CTC levels correlated with advanced clinical stage and could effectively predict PM in gastric cancer. Furthermore, preoperative FR+ CTC levels could predict the prognosis of gastric cancer patients after surgery, with a high CTC level indicating a risk of early relapse and a shorter RFS.

Gastric cancer is a leading cause of cancer-related death worldwide. PM is a common pattern of metastasis in advanced gastric cancer and is associated with poor prognosis. Due to difficulties in diagnosis and detecting techniques, synchronous PM was found during intended radical open or laparoscopic gastrectomy (27). Researchers have been working on exploring novel biomarkers and developing a nomogram to effectively predict the probability of PM. The common method to diagnose PM was conventional CT examination, with a relatively high accuracy and specificity while having a low sensitivity. Studies have shown that the sensitivity and specificity of preoperative CT diagnosis of PM in gastric cancer vary from 13% to 50.9% and from 96.2% to 99%, respectively (28–30). Modern advances in radiology equipment and computer science were promising in PM diagnosis. Chen et al. (31) developed and validated in a recent study a dual-energy computed tomography (DECT) based on the radiomics model to effectively predict PM in gastric cancer patients, with a relatively high sensitivity (53.5%, 95% CI, 37.8%–68.5%) and specificity (93.2%, 95% CI, 86.6%–96.8%). Another large-scale study also proved the value of CT-based radiomics signature in PM prediction. The radiomics prediction model was also validated in internal and external validation cohorts, with the AUCs as 0.870 (95% CI, 0.795–0.946) and 0.815 (95% CI, 0.763–0.867) (32). Besides radiology examination, several studies have also demonstrated the clinicopathological characteristics such as T stage and lymph node metastasis, and serum biomarkers including CEA, CA19-9, CA125, and CA72-4 were related to PM in gastric cancer. Emoto et al (33), showed that CA72-4, CA19-9, and CA125 were significantly positively correlated with gastric cancer with PM, with 36%, 45%, and 46% sensitivity in diagnosis respectively. Huang et al. (34) combined tumor biomarkers CA125 and CA19-9 and inflammatory markers such as fibrinogen-to-lymphocyte ratio (FLR) to construct a nomogram for risk assessment of PM in gastric cancer. The combined model showed high diagnostic sensitivity (77.4%) and specificity (94.0%). Court et al. (35) investigated CTCs as a preoperative predictor of occult metastasis in pancreatic cancer and showed that CTCs were a potential preoperative biomarker for identifying pancreatic cancer patients at high risk of OPM. In the present study, we found that gastric cancer patients with OPM had a significantly higher serum FR+ CTC level than their counterparts. The combined model of CTCs, CA19-9, prealbumin, and peripheral lymphocyte count was efficient in predicting PM (**Figure 3**), suggesting that preoperative serum FR+ CTCs could serve as a non-invasive biomarker for predicting OPM in gastric cancer. The main mode of PM is peritoneal dissemination, while CTCs mainly contribute to the hematogenous metastasis. A recent study showed the value of FR+ CTC level in predicting lymph node metastasis in patients with lung adenocarcinoma (36).

Early recurrence is common and associated with poor prognosis in gastric cancer after curative gastrectomy (5, 6). Gastric cancer early recurrence is usually defined as tumor relapse within 12–24 months after gastrectomy (37, 38). Increased risk of gastric cancer recurrence has been found to be associated with multiple factors including clinicopathological factors such as tumor size, lymph node metastasis, Lauren histologic type, lymphatic invasion, neural invasion, and elevated serum CA19-9 levels (39–41). Studies have shown that preoperative CTC levels were closely related to disease relapse in NSCLC after curative resection (42–44). Studies on gastric cancer also suggested that CTCs in peripheral blood may be a useful tool for predicting recurrence, long-term survival, and the effect of chemotherapy (45–47). These studies used different techniques to detect CTC levels. In the current study, we used the LT-PCR technique to identify FR+ CTCs in peripheral blood samples from gastric cancer patients. This two-step (negative enrichment and LT-PCR amplification) method has been validated as a sensitive method for CTC detection in different malignancies (12, 15, 22, 48).

FRs express low to negligible levels in normal tissue (49). Therefore, 9 patients with benign disease (as control) in this study also had detectable CTC with the average FR+ CTC level as 9.19 ± 1.13 FU/3 ml. Compared to benign disease, gastric cancer patients had a significantly higher FR+CTC level (12.49 ± 1.78 FU/3 ml, p < 0.001). This indicated that the FR+ CTC level has a valuable role in gastric cancer diagnosis. Meanwhile, we investigated the correlation between FR+ CTC level and traditional clinicopathologic characteristics in gastric cancer. We found that preoperative FR+ CTC level positively correlated with poor prognostic factors as CEA, CA19-9 level, lymph node metastasis, PM, and advanced TNM stage (**Table 2**). To exclude the interaction of various factors, we performed multivariate analysis for RFS and for the first time demonstrated that preoperative FR+ CTC was an independent prognostic factor for early recurrence and short RFS in gastric cancer patients after curative gastrectomy (**Table 4**). The results suggested that preoperative CTC level monitoring is promising for the treatment and follow-up plan. For patients with a high preoperative CTC level, postoperative CTC level test may also be necessary for treatment evaluation. According to the manufacturer’s instructions (15, 19), the reference range of FR+ CTCs to distinguish lung cancer from benign lung disease was 8.7 FU/3 ml. FR+ CTC is a continuous variable. The normal range may vary in different types of cancer and population. Until now, no study has reported the FR+ CTC level in gastric cancer patients. In our study, only 2 patients with gastric cancer had a <8.7 FU/3 ml FR+ CTC level (data not shown). Therefore, we did not further analyze the prognostic value of negative FR+ CTCs.

Micrometastases, beyond the surgical resection and lymphadenectomy, may contribute to the development of recurrence or metastasis after radical operation. CTCs released from micrometastasis sites played an important role in disease relapse and progression (50). Studies have shown that CTCs have good prospects of application in monitoring the efficacy of treatments such as chemotherapy, radiotherapy, and surgery (51–53). Interestingly, a recent study investigated and showed that CTC level may predict the efficacy of pemetrexed-based chemotherapy in patients with non-squamous non-small cell lung cancer (53). The author divided patients into negative CTC group (FR+ CTCs <11 FU/3 ml), low CTC group (FR+ CTCs = 11–16 FU/3 ml), and high CTC group (FR+ CTCs >16 FU/3 ml). The results showed that high CTC group achieved superior objective response rate and better prognosis, followed by negative CTC group and low CTC group. A potential mechanism may be that the inhibition of thymidylate synthase (TS) by pemetrexed was profound in the tumor cells with high expression of FR. This finding suggested that besides a simple predictive biomarker, monitoring the dynamics of FR+ CTC levels may be useful in precisely evaluating the efficacy of different treatments.

Besides CTC, CTC clusters are important groups of tumor cells that travel together in the bloodstream and, similar to CTC, contributing to the recurrence and distant metastasis in various cancers (54). The presence of CTC clusters was also proven to be a poor prognostic factor in lung cancer, breast cancer, and colorectal cancer patients (55–57). The most widely reported and the only Food and Drug Administration (FDA)-approved technique to detect CTC and CTC cluster level was CellSearch™ System. CellSearch™ System (Menarini Silicon Biosystems Inc., Huntingdon Valley, PA, USA) is an antibody-based marker-dependent platform that applied immunomagnetic separation and flow cytometry protocol to detect CTCs in peripheral blood (58). No studies directly compare the sensitivity and specificity of different CTC detection methods. The ligand-targeted enzyme-linked polymerization FR+ CTC detection was a novel and efficient method with theoretically high sensitivity. However, further studies were needed to verify the application of FR+ CTC detection in early cancer detection and outcome prediction.

### Clinical Implication and Limitations

This study provided notable points and clinical feasibility of FR+ CTCs in gastric cancer patients after laparoscopic gastrectomy. First, we discovered that preoperative FR+ CTC levels might serve as a useful diagnostic biomarker for OPM. Monitoring of FR+ CTC levels might help physicians to identify patients with OPM who might require preoperative chemotherapy. Second, we found that preoperative CTC levels can predict the short-term prognosis of gastric cancer patients who underwent radical operation, with a high FR+ CTC level indicating shorter RFS. Postoperative CTC level monitoring might be the next research focus.

We acknowledge that the present study has several limitations. First, this is a small-scale single institution-based study, with a relatively small sample size. External validation cohort is necessary for further investigation. Second, the FR+ CTC level is quantitative and can be used as a biomarker similar to tumor marker. However, the cutoff value of FR+ CTC level may vary according to different types of cancers. Therefore, the cutoff value of this study may not be suitable for other study populations. Third, the follow-up duration of this study is relatively short. The follow-up duration of some included patients did not reach 12 months (definition of early recurrence in this study). Furthermore, not available data of overall survival was acquired. To overcome these hurdles, prospective trials with a larger sample size, more clinicopathologic measurements, and longer observation period are needed to validate the findings of our study.

## Conclusions

In summary, our study for the first time suggested that detecting preoperative FR+ CTC level was useful in predicting PM and early recurrence in gastric cancer patients. Second, FR+ CTCs can be easily measured and assist the physician in identifying patients with a high risk of recurrence for close monitoring and intensive treatment.

## Data Availability Statement

The raw data supporting the conclusions of this article will be made available by the authors without undue reservation.

## Ethics Statement

The studies involving human participants were reviewed and approved by Tongji Hospital Medical Ethics Committee. The patients/participants provided their written informed consent to participate in this study.

## Author Contributions

All authors participated in the study design. Ci Dian Dan Zeng: Data curation, methodology, and software. Cheng Cheng Jin: Data curation and writing—original draft. Chun Gao: Conceptualization and writing—original draft. Ai Tang Xiao: formal analysis and project administration. Yi Xin Tong: Formal analysis and project administration. Sheng Zhang: Conceptualization and writing—review and editing. All authors have agreed on the final version and meet the major criteria recommended by the ICMJE (http://www.icmje.org/).

## Funding

This study was supported by grants from the Chinese Society of Clinical Oncology (no. Y-sy2018-029).

## Conflict of Interest

The authors declare that the research was conducted in the absence of any commercial or financial relationships that could be construed as a potential conflict of interest.

## Publisher’s Note

All claims expressed in this article are solely those of the authors and do not necessarily represent those of their affiliated organizations, or those of the publisher, the editors and the reviewers. Any product that may be evaluated in this article, or claim that may be made by its manufacturer, is not guaranteed or endorsed by the publisher.

## Supplementary Material

Supplementary Figure 1 Comparison of recurrence free survival gastric cancer patients based on preoperative FR+CTCs and CA19-9 levels. Group A: patients without elevated FR+CTCs (≥12.6FU/3mL) and elevated CA19-9 (>34 ng/mL); Group B: patients with either elevated FR+CTCs (≥12.6FU/3mL) or elevated CA19-9 (>34 ng/mL); Group C: patients with both elevated FR+CTCs (≥12.6FU/3mL) and elevated CA19-9 (>34 ng/mL).
