# Supplementary figures and images for: Preoperative Folate Receptor-Positive Circulating Tumor Cells Are Associated With Occult Peritoneal Metastasis and Early Recurrence in Gastric Cancer Patients: A Prospective Cohort Study
Source: Front Oncol. 2022 Mar 29;12:769203. doi: 10.3389/fonc.2022.769203 (PMC9002093; doi:10.3389/fonc.2022.769203)

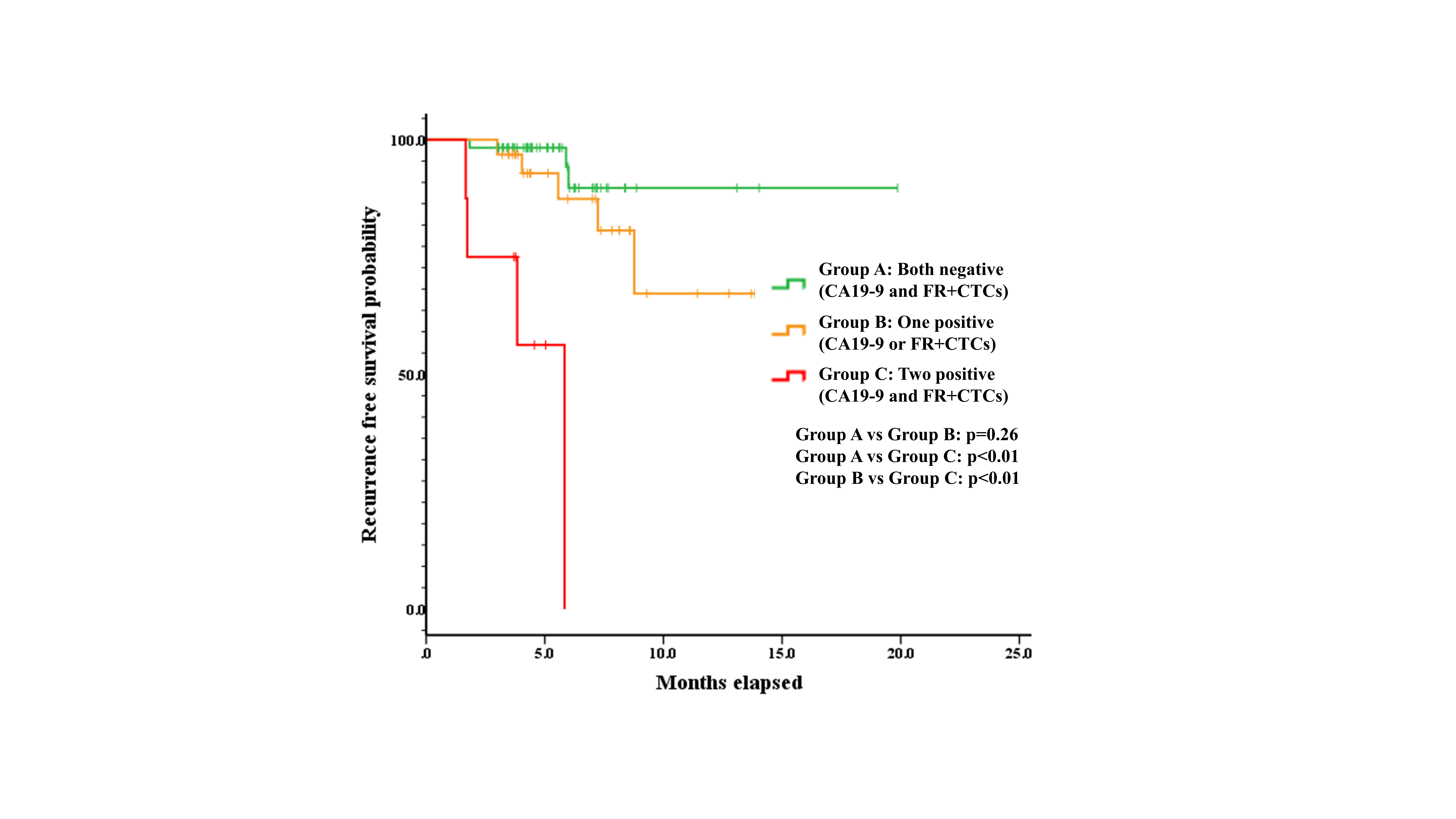

Supplement: Supplementary Figure 1 — Comparison of recurrence free survival gastric cancer patients based on preoperative FR+CTCs and CA19-9 levels. Group A: patients without elevated FR+CTCs (≥12.6FU/3mL) and elevated CA19-9 (>34 ng/mL); Group B: patients with either elevated FR+CTCs (≥12.6FU/3mL) or elevated CA19-9 (>34 ng/mL); Group C: patients with both elevated FR+CTCs (≥12.6FU/3mL) and elevated CA19-9 (>34 ng/mL). [file Image_1.tif]
