# Supplementary material for: Preoperative Folate Receptor-Positive Circulating Tumor Cells Are Associated With Occult Peritoneal Metastasis and Early Recurrence in Gastric Cancer Patients: A Prospective Cohort Study
Source: Front Oncol. 2022 Mar 29;12:769203. doi: 10.3389/fonc.2022.769203 (PMC9002093; doi:10.3389/fonc.2022.769203)
Supplement: Supplementary file 3 [file Table_2.docx]

Supplemental Table 2. Pathological characteristics of included all patients

| **Variable** | **All Patients** |
| --- | --- |
| Benign disease  Peptic ulcer  Gastric neurofibromas  Gastric adenocarcinoma | 9  8 (88.9%)  1 (11.1%)  132 |
| Tumor size, cm, mean±SD | 3.5±1.9 |
| Differentiation  High/moderate  Poor | 62 (47.0%)  70 (53.0%) |
| Signet-ring cell carcinoma  No  Yes | 95 (84.1%)  18 (15.9%) |
| Tumor deposit  No  Yes | 103 (86.6%)  16 (13.4%) |
| Vessel carcinoma embolus  No  Yes | 100 (85.5%)  17 (14.5%) |
| Neural invasion  No  Yes | 85 (72.6%)  32 (27.4%) |

SD, standard deviation
