# Supplementary material for: Preoperative Folate Receptor-Positive Circulating Tumor Cells Are Associated With Occult Peritoneal Metastasis and Early Recurrence in Gastric Cancer Patients: A Prospective Cohort Study
Source: Front Oncol. 2022 Mar 29;12:769203. doi: 10.3389/fonc.2022.769203 (PMC9002093; doi:10.3389/fonc.2022.769203)
Supplement: Supplementary file 4 [file Table_3.docx]

Supplemental Table 3. Univariate logistic regression and multivariate analysis of risk factors associated with peritoneal metastasis.

| Variable | **OR (95%CI)** | **P value** | **OR (95%CI)** | **P value** |
| --- | --- | --- | --- | --- |
| FR+ CTCs | 1.463 (1.049-2.039) | **0.025** | 1.473 (1.046-2.047) | **0.025** |
| CEA | 1.001 (0.993-1.01) | 0.762 |  |  |
| CA19-9 | 1.003 (0.999-1.006) | **0.097** | N/A | 0.189 |
| CA72-4 | 1.005 (1.00-1.01) | **0.045** | N/A | 0.056 |
| Albumin | 0.930 (0.79-1.094) | 0.382 |  |  |
| Prealbumin | 0.981 (0.964-0.997) | **0.023** | N/A | 0.126 |
| Peripheral lymphocyte count | 0.346 (0.09-1.397) | 0.136 |  |  |

For p-value: Boldface type indicates significant difference.

OR, odd ratio; N/A, not applicable; FR+ CTCs, Folate receptor positive circulating tumor cells; CEA, carcinoembryonic antigen. CA19-9, Carbohydrate antigen 19-9; CA72-4, Carbohydrate antigen 72-4;
