# Supplementary material for: Preoperative Folate Receptor-Positive Circulating Tumor Cells Are Associated With Occult Peritoneal Metastasis and Early Recurrence in Gastric Cancer Patients: A Prospective Cohort Study
Source: Front Oncol. 2022 Mar 29;12:769203. doi: 10.3389/fonc.2022.769203 (PMC9002093; doi:10.3389/fonc.2022.769203)
Supplement: Supplementary file 5 [file Table_4.docx]

Supplemental Table 4. The details of follow-up data in included patients

| **Patient ID** | **Recurrent free survival, months** | **Recurrence** | **FR+ CTCs level** |
| --- | --- | --- | --- |
| 3 | 13.10 | No | Negative |
| 6 | 8.87 | No | Negative |
| 7 | 7.67 | No | Negative |
| 9 | 8.37 | No | Negative |
| 10 | 8.40 | No | Negative |
| 13 | 7.60 | No | Negative |
| 16 | 7.37 | No | Negative |
| 18 | 7.20 | No | Negative |
| 19 | 7.20 | No | Negative |
| 20 | 7.20 | No | Negative |
| 23 | 7.13 | No | Negative |
| 24 | 7.03 | No | Negative |
| 26 | 7.00 | No | Negative |
| 31 | 6.30 | No | Negative |
| 32 | 6.30 | No | Negative |
| 34 | 6.23 | No | Negative |
| 35 | 6.23 | No | Negative |
| 36 | 6.43 | No | Negative |
| 40 | 6.03 | No | Negative |
| 42 | 5.97 | No | Negative |
| 44 | 5.97 | No | Negative |
| 45 | 5.73 | No | Negative |
| 47 | 5.63 | No | Negative |
| 49 | 5.60 | No | Negative |
| 50 | 5.60 | No | Negative |
| 52 | 5.50 | No | Negative |
| 53 | 5.37 | No | Negative |
| 54 | 5.33 | No | Negative |
| 56 | 5.13 | No | Negative |
| 58 | 5.10 | No | Negative |
| 59 | 5.10 | No | Negative |
| 61 | 4.80 | No | Negative |
| 62 | 4.67 | No | Negative |
| 64 | 19.87 | No | Negative |
| 65 | 6.00 | Yes | Negative |
| 66 | 13.70 | No | Negative |
| 68 | 14.03 | No | Negative |
| 70 | 13.87 | No | Negative |
| 71 | 12.77 | No | Negative |
| 77 | 5.90 | Yes | Negative |
| 79 | 8.57 | No | Negative |
| 82 | 4.47 | No | Negative |
| 83 | 4.47 | No | Negative |
| 86 | 4.43 | No | Negative |
| 87 | 4.40 | No | Negative |
| 89 | 4.33 | No | Negative |
| 90 | 4.27 | No | Negative |
| 91 | 4.27 | No | Negative |
| 92 | 4.23 | No | Negative |
| 94 | 4.27 | No | Negative |
| 95 | 4.20 | No | Negative |
| 97 | 4.10 | No | Negative |
| 100 | 3.73 | No | Negative |
| 101 | 3.73 | No | Negative |
| 102 | 1.83 | Yes | Negative |
| 106 | 3.73 | No | Negative |
| 110 | 3.67 | No | Negative |
| 111 | 3.63 | No | Negative |
| 114 | 3.50 | No | Negative |
| 115 | 3.47 | No | Negative |
| 116 | 3.47 | No | Negative |
| 118 | 20.00 | No | Negative |
| 119 | 3.40 | No | Negative |
| 120 | 3.40 | No | Negative |
| 121 | 3.27 | No | Negative |
| 122 | 3.27 | No | Negative |
| 124 | 3.23 | No | Negative |
| 125 | 3.20 | No | Negative |
| 127 | 3.07 | No | Negative |
| 129 | 3.03 | No | Negative |
| 130 | 3.03 | No | Negative |
| 132 | 3.83 | No | Negative |
| 1 | 7.83 | No | Positive |
| 2 | 3.00 | Yes | Positive |
| 5 | 9.30 | No | Positive |
| 8 | 8.60 | No | Positive |
| 12 | 8.13 | No | Positive |
| 15 | 4.03 | Yes | Positive |
| 17 | 7.37 | No | Positive |
| 22 | 7.13 | No | Positive |
| 25 | 7.00 | No | Positive |
| 27 | 5.83 | Yes | Positive |
| 28 | 4.93 | Yes | Positive |
| 29 | 1.73 | Yes | Positive |
| 30 | 3.83 | Yes | Positive |
| 33 | 6.27 | No | Positive |
| 43 | 5.97 | No | Positive |
| 57 | 5.13 | No | Positive |
| 60 | 5.03 | No | Positive |
| 63 | 4.57 | No | Positive |
| 67 | 5.57 | Yes | Positive |
| 69 | 8.77 | Yes | Positive |
| 73 | 11.43 | No | Positive |
| 76 | 7.23 | Yes | Positive |
| 80 | 8.13 | No | Positive |
| 85 | 4.40 | No | Positive |
| 88 | 4.37 | No | Positive |
| 93 | 4.27 | No | Positive |
| 96 | 4.10 | No | Positive |
| 99 | 3.87 | No | Positive |
| 104 | 3.77 | No | Positive |
| 105 | 3.77 | No | Positive |
| 107 | 3.70 | No | Positive |
| 108 | 3.70 | No | Positive |
| 109 | 1.67 | Yes | Positive |
| 112 | 3.63 | No | Positive |
| 117 | 3.47 | No | Positive |
| 126 | 3.20 | No | Positive |

Note: For FR+CTC level: negative indicated FR+CTC<12.6 FU/3mL, positive indicated FR+CTC≥12.6 FU/3mL.
